# Cloning of canine Ku80 and its localization and accumulation at DNA damage sites

**DOI:** 10.1002/2211-5463.12311

**Published:** 2017-11-02

**Authors:** Manabu Koike, Yasutomo Yutoku, Aki Koike

**Affiliations:** ^1^ National Institute of Radiological Sciences National Institutes for Quantum and Radiological Science and Technology Chiba Japan

**Keywords:** canine, companion animal, DNA double‐strand break, Ku70, nonhomologous DNA‐end joining

## Abstract

Molecularly targeted therapies have high specificity and significant cancer‐killing effect. However, their antitumor effect might be greatly diminished by variation in even a single amino acid in the target site, as it occurs, for example, as a consequence of SNPs. Increasing evidence suggests that the DNA repair protein Ku80 is an attractive target molecule for the development of next‐generation radiosensitizers for human cancers. However, the localization, post‐translational modifications (PTMs), and complex formation of Ku80 have not been elucidated in canines. In this study, for the first time, we cloned, sequenced, and characterized canine Ku80 cDNA. Our data show that canine Ku80 localizes in the nuclei of interphase cells and is quickly recruited at laser‐induced double‐strand break sites. Comparative analysis shows that canine Ku80 had only 82.3% amino acid identity with the homologous human protein, while the nuclear localization signal (NLS) in human and canine Ku80 is evolutionarily conserved. Notably, some predicted PTM sites, including one acetylation site and one sumoylation site within the NLS, are conserved in the two species. These findings suggest that the spatial and temporal regulation of Ku80 might be conserved in humans and canines. However, our data indicate that the expression of Ku80 is considerably lower in the canine cell lines examined than in human cell lines. These important findings might be useful to better understand the mechanism of the Ku80‐dependent DNA repair and for the development of potential next‐generation radiosensitizers targeting common targets in human and canine cancers.

**Abbreviations**
APLF: aprataxin and polynucleotide kinase/phosphatase‐like factor
DSBs: DNA double‐strand breaks
HR: homologous recombination
NHEJ: nonhomologous DNA‐end joining
NLS: nuclear localization signal
PTMs: post‐translational modifications

About 6 million dogs are diagnosed with cancer each year, and in USA, more than half of the dogs older than 10 years will develop cancers such as osteosarcoma, lymphoma, or melanoma 1. Therefore, to develop new therapies and therapeutic drugs for canine cancer is a compelling problem. Generally, mouse models are used to develop novel cancer therapies and therapeutic drugs, but their success rates is very low: Only 11% of cancer drugs that appear to be promising in mouse models turn out to be safe and effective in humans 1. Naturally developing cancers in human and canine share many characteristics 2. Therefore, canines have been considered an excellent model for basic cancer research, and for the development of new therapeutic drugs and therapies including next‐generation chemoradiotherapies for both humans and canines 1, 2, 3.

Resistance to chemotherapy and radiotherapy is a common problem in the treatment for cancers of human and companion animal such as canines. Indeed, the ability of cancer cells to repair therapeutically induced DNA damage affects the efficacy of treatments 4. DNA double‐strand breaks (DSBs) are the most harmful among all forms of DNA damage 4, 5. There are two major DSB repair pathways: nonhomologous DNA‐end joining (NHEJ) and homologous recombination (HR) 4, 5, 6. In human and rodent cells, the NHEJ pathway can be employed to repair DSBs throughout the cell cycle and is functional in both normal and cancer cells 4, 5, 6. The repair of DSBs via NHEJ is initiated by the binding of the Ku70 and Ku80 heterodimer (called Ku) to the damaged ends. At the break sites, Ku works as a critical DNA repair protein, recognizing the DSBs, protecting them against nucleolytic degradation, and recruiting other core NHEJ factors 5, 6. The critical function of Ku in NHEJ raises the possibility that targeting of Ku might sensitize cancer cells to the effect of X‐ray, heavy ion radiation, and traditional chemotherapeutics.

To develop novel molecularly targeted anticancer drugs for both humans and canines, it is essential to determine target amino acids (and their involvement in protein structure) in a specific molecule of the chosen pathway. The recruitment to DSBs, protein–protein interactions, and post‐translational modifications (PTMs) of Ku80 might play critical roles in the regulation of NHEJ activity. Previously, we have shown that Ku80 has a nuclear localization signal (NLS) recognized by NLS receptors and is mainly localized in the nucleus during interphase in various human cell lines 6, 7, 8. Furthermore, the localization of human Ku80 is regulated through the cell cycle 6, 7. Moreover, it was demonstrated that the human Ku80 is recruited to DSB sites immediately after laser irradiation 5, 9. On the other hand, it has not been clarified whether Ku80 of other species including canines is recruited to DSB sites immediately after DNA damage. Human Ku80 might be one charming target for the development of next‐generation radiosensitizers 4, 10, 11. Meanwhile, canine *Ku80* cDNA has not been cloned until now. In addition, the sequence, localization, and control mechanisms of canine Ku80 have not been published.

In this study, we first cloned *Ku80* cDNA from a canine testis library and performed comparative analysis to clarify the regulatory mechanisms of Ku80 functions. Furthermore, we investigated its expression, subcellular localization, and recruitment to DSB sites.

## Materials and methods

### Cloning of canine Ku80

The primers used to amplify canine *Ku80* cDNA from a male beagle dog cDNA library (Biochain, Newark, CA, USA) were designed based on the predicted *Ku80* genomic sequence of female boxer dog, belonging to the species *Canis lupus familiaris* (XM_536061.3). The primers used for PCR and sequencing are listed in Table 1. PCR amplification with sense (F) and antisense (R) primers was performed in a thermal cycler using the Takara Tks Gflex DNA polymerase (Takara Bio Inc., Otsu, Japan). Predenaturation was carried out for 1 min at 94 **°**C, and it was followed by 30 cycles of PCR amplification. Each cycle consisted of denaturation at 98 **°**C for 10 s, annealing at 55 **°**C for 15 s, and extension at 68 **°**C for 1.5 min. PCR products were subcloned into the *Eco*RI and *Apa*I sites of the pEYFP‐C1 plasmid (pEYFP‐canine *Ku80*) using the In‐Fusion HD cloning kit (Takara Bio Inc.). The inserts were validated by sequencing using sequencing primers, dX5 P1, dX5 P2, dX5 P6, and dX5 P7 (Table 1). PCR amplification with sense (dX5 Cseq F2 or dX5 Nseq F2) and antisense (dX5 Cseq R2 or dX5 Nseq R1) primers was carried out for 35 cycles in a Thermal Cycler Dice (Takara Bio Inc.) or a Thermal Cycler PC‐700 (ASTEC, Fukuoka, Japan) using LA Taq polymerase (Takara Bio Inc.). After predenaturing (95 **°**C for 2 min or 94 **°**C for 5 min), each cycle consisted of denaturation at 94 **°**C for 1 min or 0.5 min, annealing at 60 **°**C for 1 min or 0.5 min and extension at 72 **°**C for 1 min or 0.5 min, followed by a final extension (4 min or 5 min). The PCR products were subcloned into pCR4‐TOPO vector (Invitrogen, Carlsbad, CA, USA), and the nucleotide sequences were determined by sequencing using sequencing primers, T3 and T7 (Table 1).

**Table 1:** Primer sequences used for PCR and sequencing

Primer	Bases	Sequence (5′–3′)
PCR primers		
Forward: F	37 bp	5 ′ ‐CTCAAGCTTCGAATTCGATGGCGGCGTCCAGGAGCAA‐3 ′
Reverse: R	45 bp	5 ′ ‐ATCCGGTGGATCCCGGGCCCCTATATCAAGTCCAGTAAATCATCC‐3 ′
Forward: dX5 Cseq F2	27 bp	5 ′ ‐GAACCTCCAAATGAAGACACAGCAGCC‐3 ′
Reverse: dX5 Cseq R2	27 bp	5 ′ ‐CACATAACCGACAGGGACCATCTCCAG‐3 ′
Forward: dX5 Nseq F2	19 bp	5 ′ ‐ACTCCCCCCGGACCTTGGC‐3 ′
Reverse: dX5 Nseq R1	28 bp	5 ′ ‐TTCTGATACTGATCCTTACCAGCAAGGG‐3 ′
Sequencing primers		
dX5 P1	21 bp	5 ′ ‐GCTGGTAAGGATCAGTATCAG‐3 ′
dX5 P2	21 bp	5 ′ ‐GACAGTTGTGGACGCTAGAAC‐3 ′
dX5 P6	21 bp	5 ′ ‐CAGTGAACACTTCAATATGTC‐3 ′
dX5 P7	21 bp	5 ′ ‐CAGAGATTATTTCAGTGTCTG‐3 ′
T3	17 bp	5 ′ ‐ATTAACCCTCACTAAAG‐3 ′
T7	17 bp	5 ′ ‐AATACGACTCACTATAG‐3 ′

### Cell lines, cultures, and transfections

The Madin–Darby canine kidney (MDCK) cell line (HSRRB, Osaka, Japan), the canine lung adenocarcinoma (CLAC) cell line (HSRRB), a mouse embryonic fibroblast cell line (NIH3T3; Riken Cell Bank, Tsukuba, Japan), a murine lung epithelial (Ku70+/−MLE) cell line, a human cervical carcinoma cell line (HeLa; Riken Cell Bank), and a human colon cancer cell line (HCT116; Riken Cell Bank) were cultured in Dulbecco's modified Eagle's medium with 10% FBS 12, 13, 14, 15. pEYFP‐canine *Ku80* or pEYFP‐C1 was transiently transfected into cells using Lipofectamine 3000 (Invitrogen). Post‐transfection, cells were cultured for 2 days and then observed under an FV300 confocal laser‐scanning microscope (Olympus, Tokyo, Japan), as previously described 8, 13, 14, 15, 16, 17, 18. DNA in the fixed cells was stained with 4,6‐diamino‐2‐phenylindole (DAPI) fluorescent dye, as previously described 18.

### Western blot analysis

The extraction of total cell proteins and western blot analysis were carried out as described previously 14, 15, 18 with the following modifications. The following antibodies were used: rabbit anti‐Ku80 polyclonal antibody against human Ku80 (AHP317; Serotec, Oxford, UK), rabbit anti‐Ku80 polyclonal antibody against canine Ku80 (583V AP), rabbit anti‐GFP polyclonal antibody (FL, Santa Cruz Biotechnology, Santa Cruz, CA, USA), and mouse anti‐β‐actin monoclonal antibody (Sigma, St. Louis, MO, USA). The anti‐Ku80 antibody (583V AP) was raised for this study against corresponding to the amino acids: 277–290 of canine Ku80, characterized and affinity‐purified. The two anti‐Ku80 and anti‐GFP antibodies were diluted in Signal Enhancer HIKARI (Nacalai Tesque, Kyoto, Japan).

### Local DNA damage induction using laser and cell imaging

Local DNA damage induction using laser and subsequent cell imaging was carried out as described previously 9, 15, 17. Immunocytochemistry was carried out using a mouse anti‐γH2AX monoclonal antibody (JBW301; Upstate Biotechnology Inc., Charlottesville, VA, USA) and an Alexa Fluor 568‐conjugated secondary antibody (Molecular Probes, Eugene, OR, USA), as previously described 14, 15, 18.

## Results

### Cloning and sequence analysis of canine Ku80

**Figure 1:**
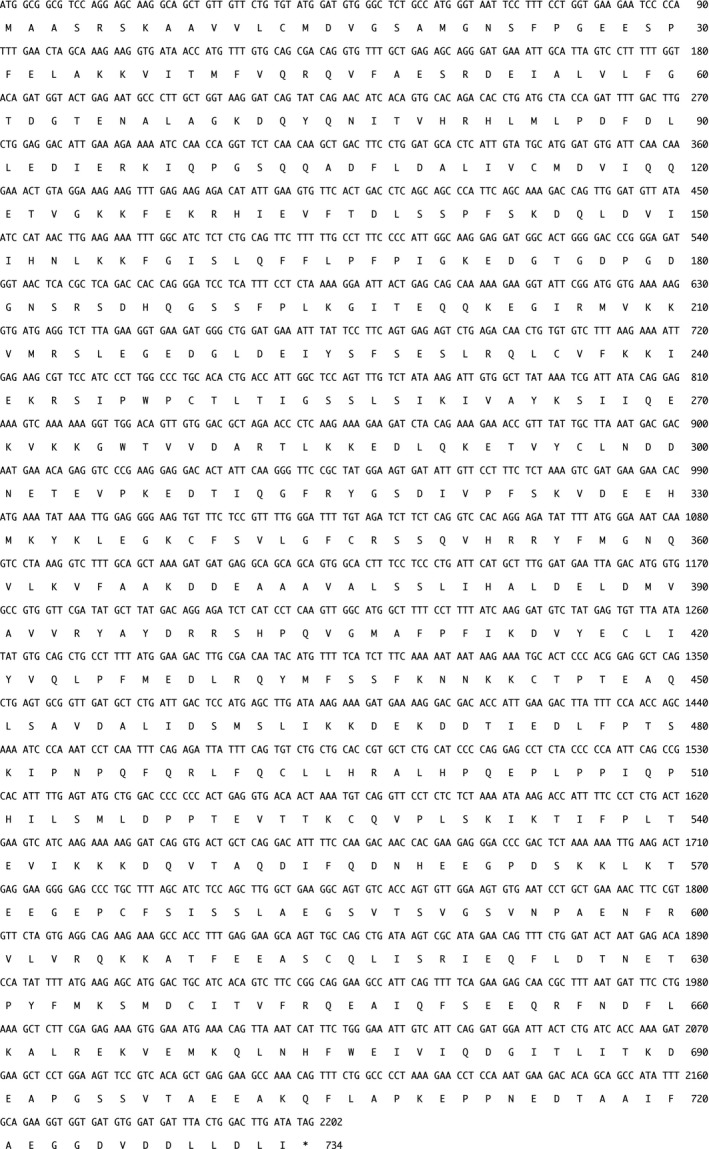
Nucleotide sequence and deduced amino acid sequences of canine Ku80 (*Canis lupus familiaris*, GenBank accession number: LC195222). The CDS of canine Ku80 is composed of 2202 bp encoding 733 amino acid residues. The asterisk after the amino acid sequence indicates the position of the termination codon. Numbers on the right correspond to nucleotides (top) and amino acid residues (bottom).

**Figure 2:**
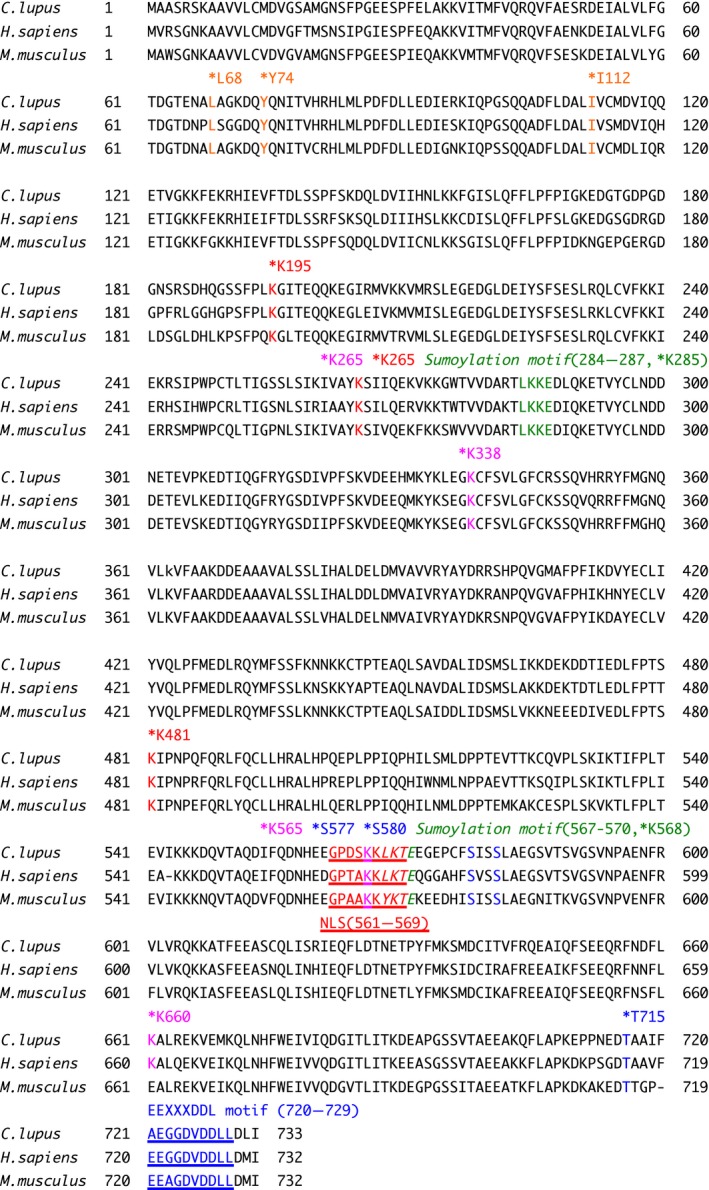
Comparison of the canine, human, and mouse Ku80 amino acid sequence. Canine (*Canis lupus familiaris*, GenBank accession number: LC195222), human (*Homo sapiens,* GenBank accession number: NP_066964.1) and mouse (*Mus musculus*, GenBank accession number: NP_033559.2) species. The location of the DNA‐PK phosphorylation sites (S577, S580, and T715), the APLF‐binding sites (L68, Y74, and I112), the ubiquitination sites (K195, K265, and K481), the acetylation sites (K265, K338, K565, and K660), and the two putative sumoylation sites (K285 and K568) in human Ku80 9, 19, 22, 24, 26, 27 are marked with asterisks. The location of the NLS (NLS: 561–569), the two putative canonical sumoylation consensus motifs [ψ‐K‐X‐E: LKKE(284–287) and LKTE(567–570)], and the EEXXXDDL motif [EEGGDVDDLL(720–729)] in human Ku80 are shown 8, 9, 22, 25.

Firstly, we cloned the canine *Ku80* cDNA from a beagle dog testis library and sequenced it. For the first time, we isolated a 2202‐nucleotide open reading frame encoding a protein of 733 amino acids (Fig. 1). The novel canine sequence has been deposited to the DDBJ/ENA/NCBI database [accession number LC195222]. Comparative analysis of Ku80 sequences from different species showed that canine Ku80 had 82.3% and 81.4% amino acid identity with the human and mouse proteins, respectively. As shown in Fig. 2, human Ku80 is modified through some PTMs, including phosphorylation, acetylation, and ubiquitination 9, 19, 20, 21, 22, 23, 24. In addition, human Ku80 has two putative sumoylation consensus motifs [ψ‐K‐X‐E: LKKE(284–287) and LKTE(567–570)] and the EEXXXDDL motif [EEGGDVDDLL(720–729)], which is a PIKK interaction motif of human Ku80 9, 22, 25. In this study, we found that the two sumoylation motifs in human Ku80 are conserved in the canine protein, whereas one of them (the motif spanning amino acids 567–570) is not conserved in mouse Ku80 (Fig. 2), as described previously 9. Contrarily, the EEXXXDDL motif in human Ku80 is conserved in mouse species and, surprisingly, not perfectly conserved in canine Ku80. We have previously demonstrated that human Ku80 has a NLS sequence spanning amino acids 561–569, which has been classified as a conventional single‐basic type sequence 6, 8. We found that this NLS motif is conserved in canine and mouse Ku80 (Fig. 2). Additionally, the location of the DNA‐PK phosphorylation sites (S577, S580, and T715), the aprataxin and polynucleotide kinase/phosphatase‐like factor (APLF)‐binding sites (L68, Y74, and I112), the ubiquitination sites (K195, K265, and K481), and the acetylation sites (K265, K338, and K565) in human Ku80 was also found to be evolutionarily conserved in both canine and mouse Ku80 (Fig. 2) 19, 24, 26, 27.

### Expression and localization of Ku80 in canine cells

**Figure 3:**
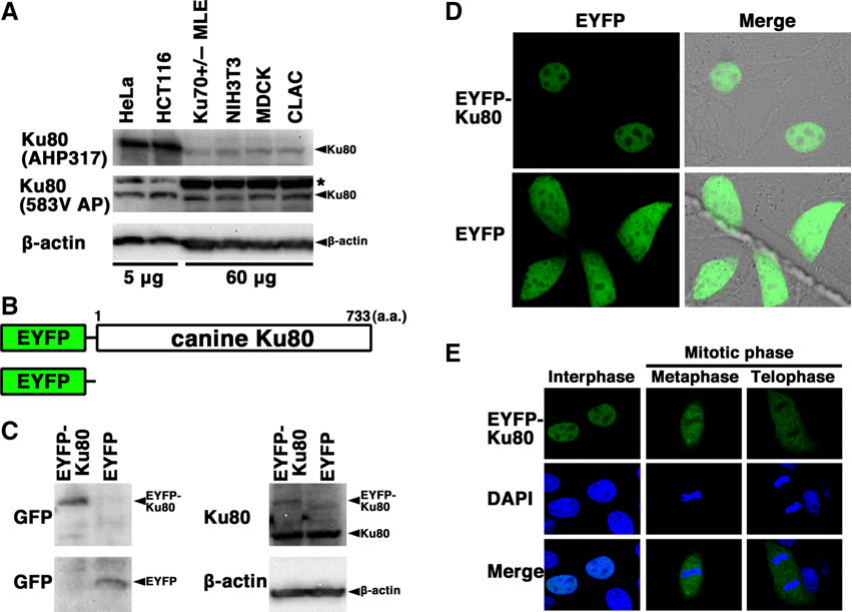
Localization and expression of canine Ku80. (A) Expression of canine Ku80. Total cell proteins from the two canine cell lines (MDCK and CLAC, 60 μg per lane), two murine cell lines (NIH3T3 and Ku70 +/− MLE, 60 μg per lane), and two human cell lines (HeLa and HCT116, 5 μg per lane) were analyzed by western blotting using an anti‐Ku80 antibody (AHP317; top), an anti‐Ku80 antibody (583V AP; middle), or an anti‐β‐actin antibody (bottom). *, nonspecific band. (B) Scheme relative to the EYFP‐canine Ku80 chimeric protein (EYFP‐canine Ku80, top) and control protein (EYFP, bottom). (C) Expression of EYFP‐canine Ku80. Extracts from MDCK cells transiently expressing EYFP‐canine Ku80 or EYFP were analyzed by western blotting using anti‐GFP, anti‐Ku80, and anti‐β‐actin antibodies. (D) Imaging of live cells transfected with pEYFP‐canine *Ku80*. Live MDCK cells transiently expressing EYFP‐canine Ku80 or EYFP were observed by confocal laser microscopy. EYFP images for the same cells are shown alone (left panel) or merged (right panel) with the corresponding differential interference contrast images. (E) Imaging of fixed cells transfected with pEYFP‐canine *Ku80*. MDCK cells transiently expressing EYFP‐canine Ku80 were fixed and stained with DAPI. The stained cells were observed by confocal laser microscopy. The images shown are a representative example for interphase cells or mitotic phase cells. Top panel, EYFP‐Ku80 image; middle panel, DAPI image; bottom panel, merged image.

Next, we analyzed the expression and subcellular localization of canine Ku80. First, we examined the expression of Ku80 in canine (MDCK and CLAC), murine (NIH3T3 and Ku70+/−MLE), and human (HeLa and HCT116) cell lines. As shown in Fig. 3A, the expression of canine Ku80 was detected by the two antibodies (AHP317 and 583V AP) in the two canine cell lines examined; similar to what observed in the murine cell lines, Ku80 displayed lower expression levels in the two canine cell lines compared to the human cell lines. Next, to investigate subcellular localization of Ku80 in live canine cells, we generated MDCK cells transiently expressing EYFP‐canine Ku80. For this purpose, the expression vector pEYFP‐C1 containing canine *Ku80* (pEYFP‐canine *Ku80*) was transfected into MDCK cells (Fig. 3B). As shown in Fig. 3C, western blotting using anti‐Ku80 and anti‐GFP antibodies showed that the chimeric protein was expressed in the transfected cells. Confocal laser microscopy demonstrated that, during interphase, EYFP‐canine Ku80 localized in the nuclei (with the exclusion of the nucleoli) of the cells transfected with pEYFP‐canine *Ku80* (Fig. 3D). Expectedly, EYFP, used as a control, was distributed throughout the cell (with the exclusion of the nucleoli) in pEYFP‐transfected cells (Fig. 3D), consistent with our previous reports 14, 15, 18. Meanwhile, during mitosis, EYFP‐canine Ku80 was detected throughout the cytoplasm of the transfected cells, but was not localized to mitotic chromosomes (Fig. 3E). These results showed that the localization of canine Ku80 dynamically changes during the cell cycle.

### EYFP‐canine Ku80 accumulates immediately at laser‐microirradiated DSB sites

**Figure 4:**
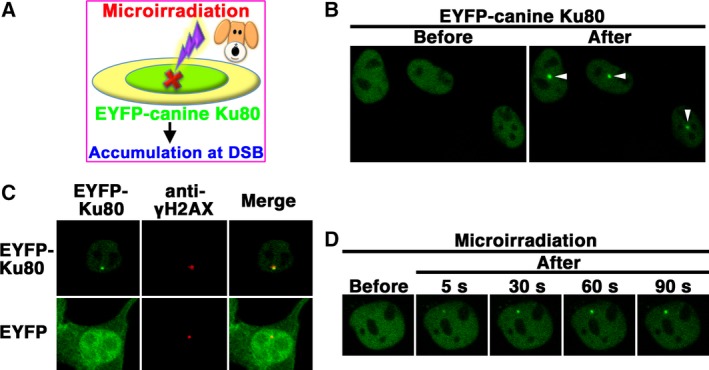
Accumulation of EYFP‐canine Ku80 at the sites of DSBs induced by laser microirradiation. (A) The recruitment of EYFP‐canine Ku80 to DSBs induced by 405‐nm laser irradiation in MDCK cells. (B) Imaging of live MDCK cells transfected with pEYFP‐canine *Ku80* before (left panel) and after (right panel) microirradiation. Arrowheads indicate the microirradiated sites. (C) Immunostaining of microirradiated cells transfected with pEYFP‐canine *Ku80* using an anti‐γH2AX antibody. Cells were fixed and stained with an anti‐γH2AX antibody 5 min postirradiation. Left panel, EYFP‐canine Ku80 (upper panel) or EYFP (lower panel); center panel, γH2AX; right panel, merged images. (D) Time‐dependent EYFP‐canine Ku80 accumulation in live cells, from 5 to 90 s after irradiation.

Previously, we and others have demonstrated that human Ku80 accumulates immediately at DSB sites after irradiation 5, 9. Meanwhile, it has not yet been investigated whether Ku80 of other species, including canine, accumulates at DSB sites immediately after DNA damage. Next, we investigated whether, in canine cells, EYFP‐canine Ku80 accumulates immediately at DSB sites induced by a 405‐nm laser (Fig. 4A). Local DSBs in canine cells were induced using a 3% power scan (for 1 s) from a 405‐nm laser. Laser microirradiation resulted in the accumulation of EYFP‐canine Ku80 at the microirradiated sites in live MDCK cells (Fig. 4B). To test whether EYFP‐canine Ku80 actually accumulated at the DSB sites induced by the 405‐nm laser, we immunostained cells with an antibody that detects γH2AX, a marker of DSBs. As shown in Fig. 4C, EYFP‐canine Ku80, but not EYFP, was found to accumulate and colocalize with γH2AX at microirradiated sites in MDCK cells. To examine the temporal dynamic of Ku80 localization, we carried out time‐lapse imaging in MDCK cells transfected with pEYFP‐canine *Ku80*. We found that EYFP‐canine Ku80 accumulates at the microirradiated sites 5 s after irradiation (Fig. 4D). These results indicate that after irradiation, EYFP‐canine Ku80 accumulates immediately and forms foci at laser‐induced DSBs in canine cells.

## Discussion

Molecularly targeted therapies have high specificity and the significant cancer‐killing effect, but their impact is known to decrease remarkably when there is a variation, such as an SNP in the target site. Comparative analysis of certain DNA repair proteins between humans and canines is useful in the attempt to develop more effective repair inhibitors and more efficient cancer‐killing drugs for both human and canine cancers. However, there is no information regarding the function and the regulation of canine Ku80. In the present study, for the first time, we cloned, sequenced, and characterized canine *Ku80* cDNA. Comparative analysis showed that canine Ku80 had only 82.3% amino acid identity with the homologue human protein. Sequence alignment demonstrated that the sites of PTMs and protein‐binding motifs are not perfectly conserved between canine Ku80 and human Ku80. Additionally, we demonstrated that EYFP‐canine Ku80 mainly localizes in the nuclei of interphase cells, and the localization of EYFP‐canine Ku80 dynamically changes during the cell cycle. Moreover, we showed that canine Ku80 is recruited to DSB sites immediately after irradiation. These findings might be useful for the development of next‐generation radiosensitizers targeting potential common targets in human and canine cancers.

Molecular mechanisms underlying protein–protein interactions and PTMs of DNA repair proteins play key roles in the control of some DNA repair pathways 5, 28. Three DNA‐PK phosphorylation sites (S577, S580, and T715) have been identified in human Ku80 20, but the role of these modifications is still unclear. In humans, Ku80 phosphorylation at DNA‐PK phosphorylation sites might not be required for its interaction with Ku70, its nuclear translocation, and DNA DSB repair 26, 29. In this study, we found that the DNA‐PK phosphorylation sites are perfectly conserved in human and canine Ku80. Further studies are necessary to clarify the significance of DNA‐PK‐mediated phosphorylation of canine Ku80, and shed light on its function and/or regulation of Ku80, not only in canines, but also in various species, including humans.

In this study, we showed that canine Ku80 accumulated at laser‐induced DSB sites immediately after irradiation. Human Ku80 is required for the recruitment of core NHEJ factors such as DNA‐PKcs, XRCC4, Ku70, and XLF 5, 9. Additionally, a study has reported that the Ku80‐binding motif of APLF promotes APLF accumulation at DSBs in human cells 27. In the same study, the authors also showed that APLF promotes the assembly and activity of core NHEJ protein complexes. In this study, our data showed that the APLF‐binding sites (L68, Y74, and I112) in human Ku80 are perfectly conserved in canines, suggesting that the interaction between APLF and Ku80 is important for the Ku's function in both species. Falck *et al*.^25^ reported that the PIKK interaction motif of human Ku80 [also called EEXXXDDL motif (720–729)] is required for the interaction with DNA‐PKcs and for DNA‐PK activation. Deletion of the last 14 residues of Ku80 (amino acids 719–732) affects the efficient recruitment of DNA‐PKcs to DNA ends *in vitro*, and point mutations (E720A/E721A) of Ku80 inhibited its interaction with DNA‐PKcs. In addition, Gell and Jackson 30 reported that the final 12 amino acid residues (amino acids: 721–732) of human Ku80 are sufficient to bind DNA‐PKcs *in vitro*. Altogether, these results suggest that the nine amino acid residues (amino acids: 721–729) of human Ku80 are sufficient to bind DNA‐PKcs. On the other hand, in this study, we found that the EEXXXDDL motif (amino acids: 720–729) of human Ku80 is not perfectly conserved in canine Ku80 (amino acids: 721–730): Canine Ku80 has an alanine residue (A721) instead of the glutamic acid residue in human Ku80. Altogether, we speculate that as well as the first glutamic acid residue (E720) of the EEXXXDDL motif in human Ku80, the corresponding alanine residue (A721) in canine Ku80 is not essential for the recruitment of DNA‐PKcs to DNA ends. Additional studies, however, are needed to clarify this issue.

The mechanism responsible for the nuclear localization of Ku70 and Ku80 appears to play, at least in part, a key role in the regulation and the physiological function of Ku *in vivo* 6. Previously, we showed that subcellular localization of human Ku80 dynamically changes during cell cycle 7. Additionally, we identified the NLS motif spanning amino acids 561–569 in human Ku80 6, 7, 8: Its structure is conserved among various species, including three rodent species and a frog 6, 8. We also clarified that human Ku80 translocates to the nucleus through the interaction between its own NLS and classical NLS receptors 8. In the present study, our data showed that the localization of canine Ku80 dynamically changes during the cell cycle. Combining with our previous findings, our data indicate that the patterns of subcellular localization of human and canine Ku80 are similar throughout the cell cycle 7. We found that the NLS motif is conserved in canine Ku80. In addition, sequence alignment analysis showed that the four acetylation sites present in human Ku80 are conserved in the canine protein, and one of these sites lies within the NLS of the three species examined. We also found that the two sumoylation consensus motifs in human Ku80 are conserved in canine Ku80, and one of these sites lies within the NLS motif as well. Collectively, these findings raise the possibility that the modifications of all or a part of these amino acids within the NLS determine the cell cycle phase‐dependent subcellular localization of Ku80, and thereby control Ku80 function. We are interested in further studies investigating this possibility.

RNF8‐, RNF138‐, and NEDD8‐dependent ubiquitin ligases have been reported to mediate human Ku80 ubiquitination 19, 21, 23. Brown *et al*. 19 reported that neddylation promotes ubiquitination and release of Ku from DNA damage sites and showed that ubiquitination of K195, K265, and K481 of human Ku80 is increased after treatment with the DSB inducer phleomycin. In this study, our data showed that these three lysines are conserved among the three species examined. Most recently, Ishida *et al*. 31 reported that RNF126 is a novel regulator of NHEJ that promotes completion of DNA repair by ubiquitinating Ku80 and releasing Ku from damaged DNA. Using proteomics and structural analyses, the authors identified 19 lysine residues including several novel ubiquitination sites in Ku80: The mutation of all of these sites inhibited the dissociation of Ku from chromatin and DNA damage response. In this study, our data showed that these 19 lysine residues in human Ku80 sites are perfectly conserved in canine Ku80, whereas three of these 19 lysine residues in human Ku80 sites, K144, K282, K469, correspond to different amino acids in mouse Ku80. Further studies need to clarify whether the ubiquitination of these three lysines has specific roles in human and canine cells. Interestingly, our findings showed that Ku80 exhibited lower expression levels in the canine cell lines compared to the human ones. The ubiquitin‐proteasome pathway can regulate the levels of certain protein via ubiquitination 32. We are interested in understanding whether the protein level of Ku80 is regulated via the ubiquitin‐proteasome pathway in human and canine cells.

In this study, we cloned, sequenced, and analyzed canine *Ku80*. Our findings provide important information for clarifying the regulation mechanism of Ku80 and its functions in canine cells. In addition, these findings might contribute to clarify the molecular mechanism of the Ku80‐dependent NHEJ pathway. Further comparative studies on Ku80 will provide valuable additional information in order to develop common molecularly targeted drugs having high specificity and significant cancer‐killing effect in both humans and canines.

## Data Accessibility

The sequence of canine Ku80 cloned in this study has been deposited to the DDBJ/ENA/NCBI database [accession number LC195222].

## Author contributions

MK and AK conducted all experiments. MK wrote the manuscript. MK, AK, and YY performed experiments and analyzed data. All authors have read and approved the final manuscript.
